# E3 Ligases and Deubiquitinases in Controlling High-Mobility Group Box (HMGB) Protein Functions

**DOI:** 10.3390/ijms27125588

**Published:** 2026-06-20

**Authors:** Elena V. Chikhirzhina, Alexey N. Tomilin, Anna S. Tsimokha

**Affiliations:** Laboratory of Molecular Biology of Stem Cells, Institute of Cytology of the Russian Academy of Sciences, Tikhoretsky Av. 4, Saint Petersburg 194064, Russia; e.chikhirzhina@incras.ru (E.V.C.); a.tomilin@incras.ru (A.N.T.)

**Keywords:** HMGB1-4 proteins, E3 ligase, deubiquitinase (DUBs), ubiquitination, ubiquitin–proteasome system

## Abstract

High-Mobility Group Box (HMGB) proteins belong to the family of high-mobility proteins characterized by two DNA-binding domains and an unstructured, negatively charged C-terminal domain that modulates DNA–protein and protein–protein interactions. These proteins participate in multiple cellular processes, including DNA replication, transcription, recombination, and repair. The functional activity of HMGB proteins is associated with various physiological and pathological conditions, including malignant tumors and cardiovascular diseases, highlighting the need for strict regulation of their levels and activity to maintain cellular homeostasis. Such regulation can occur at multiple levels, including proteolytic degradation. In recent years, a number of E3 ubiquitin ligases that promote the degradation of HMGB proteins, as well as deubiquitinases (DUBs) that stabilize them by removing ubiquitin tags, have been identified. This review summarizes these enzymes and their proposed roles in controlling the functions of the HMGB family proteins, both through direct interaction with these proteins and via mediator proteins.

## 1. Introduction

High-mobility group box (HMGB) proteins belong to the high-mobility group (HMG) superfamily [1]. The HMG family is divided into three main subgroups—HMGA, HMGB, and HMGN—each characterized by functionally significant structural domains [2]. HMGA proteins contain the AT-hook motif that binds AT-rich DNA-regions with low sequence specificity. HMGN proteins possess nucleosome-binding domains and specifically recognize the overall nucleosome structure. These proteins stabilize chromatin and modulate its epigenetic plasticity. Proteins of the HMGB family contain two structurally conserved DNA-binding domains, Box A and Box B, connected by a flexible linker, as well as short N-terminal and long C-terminal regions (reviewed in [3,4,5]). The C-terminal region consists of an unstructured, negatively charged segment enriched in Asp and Glu residues. HMGB protein sequences share over 80% identity, with the major difference residing in the length of the acidic C-terminal domain: HMGB1 contains 30, HMGB2 contains 22, and HMGB3 contains 20 residues, whereas HMGB4 lacks this region entirely [6]. It is hypothesized that the unstructured C-terminal region modulates HMGB interaction with other proteins and nucleic acids [7,8,9].

The function of HMGB proteins is strongly influenced by cellular localization (nuclear, cytoplasmic, or extracellular) [3,4,5]. Nuclear–cytoplasmic shutting is regulated by nuclear localization signals (NLS), nuclear export signals (NES), as well as by specific post-translational modifications (PTMs) [10]. It is known that PTMs such as acetylation, phosphorylation, methylation, ADP-ribosylation, and the redox state of HMGB proteins primarily affect the transport of proteins within the cell and their exit into the extracellular space [11]. In addition, PTMs affect the interaction of HMGB proteins with other proteins and with DNA, as well as the binding of HMGB proteins to membrane receptors [12]. It is important to note that the intracellular distribution of HMGB proteins, characteristic of normal physiological conditions, can change under stress or pathological conditions [13].

In the nucleus, HMGB proteins perform a regulatory function and, along with histones, are essential for the proper structural organization of chromatin [14,15]. HMGB proteins induce bending of the DNA minor groove toward the major groove, which facilitates access for regulatory proteins, including transcription factors, thereby enhancing their functional activity [16]. These proteins thus play a key role in regulating such fundamental DNA-dependent processes as transcription, repair, recombination, and replication. Overall, HMGB proteins have high affinity for structurally distorted DNA regions [17], including adducts formed by platinum-based chemotherapeutics, such as cisplatin, the formation of which induces tumor cell apoptosis. Binding of HMGB proteins to such damaged DNA regions prevents access of repair complexes to the damage site, which influences the cellular response to DNA damage and the effectiveness of anticancer drugs [18].

HMGB proteins also bind RNA either directly or through other RNA-binding proteins, suggesting roles in RNA metabolism (reviewed in [19]). In turn, regulation of HMGB proteins by various non-coding RNAs, such as lncRNA and microRNA, has also been demonstrated [20,21,22]. It is believed that dysregulation of HMGB-RNA interactions may contribute to disease development. However, the precise RNA-binding determinants and structural consequences of HMGB-RNA interactions remain insufficiently characterized. Of note, HMGB proteins interact with nucleic acids, including RNA, in a structure-dependent rather than sequence-specific manner [19].

Stress or infection can disrupt the typical HMGB nuclear localization pattern. In the cytoplasm, HMGB proteins play a key role in regulating stress and cell survival, primarily by promoting autophagy. For example, it has been shown that HMGB1 interacts with Beclin-1 (BECN1), a key autophagy regulator, thereby enhancing this process [23].

The release of HMGB proteins from the cell occurs upon loss of cell integrity during cell death modalities such as apoptosis, pyroptosis, or necrosis [13,24]. In the extracellular space, HMGB1 acts as an alarmin, a signaling molecule that is produced under inflammatory conditions to trigger immune cells and is capable of accelerating tissue regeneration and repair [12]. The extracellular functions of HMGB2 and HMGB3 remain less well characterized, although HMGB2 shows lower affinity for membrane receptors and reduced pro-inflammatory activity compared to HMGB1 [25].

HMGB proteins participate not only in DNA-associated processes such as chromatin organization, transcription, and repair, but also in non-genomic pathways, including inflammatory signaling, immune responses, autophagy, and cell death. Consequently, their dysregulation is implicated in a broad spectrum of pathological conditions. While their overexpression and role in promoting tumor cell proliferation, metastasis, and therapy resistance are well documented in various cancers [26], HMGB proteins are equally critical in non-malignant pathologies. Beyond cancer, they contribute to the pathogenesis of neurodegenerative disorders, such as Parkinson’s disease, Alzheimer’s disease, epilepsy, and amyotrophic lateral sclerosis via RAGE- and TLR4-dependent signaling [27,28]. Altered HMGB expression has also been linked to metabolic and autoimmune disorders, including type 1 and type 2 diabetes and systemic lupus erythematosus, through modulation of Akt/mTOR, MAPK, ERK1/2, and NF-κB pathways associated with autophagy (reviewed in [29]). In cardiovascular disease, reduced HMGB levels in heart failure may be associated with cardiomyocyte hypertrophy and fibrosis, whereas HMGB1 release from cardiomyocytes promotes neutrophil extracellular trap formation, contributing to cardiac remodeling and dysfunction [30,31,32]. These diverse roles underscore the importance of tight control of HMGB protein abundance and activity to maintain cellular homeostasis across all physiological and pathological contexts. This regulation is achieved at multiple levels, including transcriptional control, post-translational modification, and proteolytic control of protein stability.

The degradation of HMGB proteins, like that of other proteins, is closely linked to ubiquitination, a key PTM that plays a crucial role in cellular processes. In addition to degradation, ubiquitination is involved in intracellular transport, mRNA translation, endocytosis, protein activation and inactivation, protein–protein interactions, and the cellular response to DNA damage [33,34].

This review focuses on E3 ubiquitin ligases and deubiquitinases (DUBs) of the ubiquitin–proteasome system (UPS), which have been implicated in the regulation of HMGB protein functions both through direct interaction with these proteins and indirectly, via protein mediators.

## 2. Regulation of HMGB Proteins’ Stability

Abnormal ubiquitin-mediated protein degradation contributes to tumor cell proliferation, migration, and therapy resistance in several cancers (reviewed in [35]). Approximately 80–90% of cellular protein degradation is mediated by UPS. The proteasome recognizes ubiquitinated proteins and utilizes them as substrates [36]. Although autophagy–lysosomal degradation is considered non-selective, it is now clear that, similar to the UPS, ubiquitin modification can also function as a degradation signal for selective autophagy [37].

**Figure 1:**
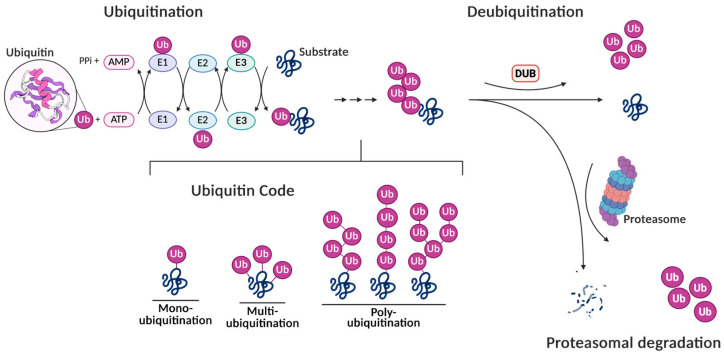
The processes of ubiquitination and deubiquitination occur within the ubiquitin–proteasome system (UPS). The process proceeds in several steps involving the ubiquitin-activating enzyme E1 (depicted in purple), the ubiquitin-conjugating enzymes E2 (blue), and the E3 ubiquitin ligases (green). E1 enzymes activate ubiquitin (Ub, shown as pink spheres) in an ATP-dependent process. E2 enzymes position ubiquitin for transfer. E3 ligases direct ubiquitin to the target protein, marking it for degradation. Deubiquitinases (DUBs, depicted in orange) remove ubiquitin chains from target proteins, allowing substrates to be recycled or spared from degradation, thereby preventing the unnecessary destruction of functional proteins. Created in BioRender. Tomilin, A. (2026) https://BioRender.com/bhkbilj (accessed on 17 June 2026).

Ubiquitination is a critical reversible PTM involving the covalent attachment of ubiquitin, a small 76-amino-acid protein, to target proteins (Figure 1). The process proceeds in several steps involving the ubiquitin-activating enzyme E1, the ubiquitin-conjugating enzymes E2, and the E3 ubiquitin ligases [38]. Whereas E2 enzymes position ubiquitin for transfer, E3 ligases provide substrate specificity by directing the transfer of ubiquitin to the target protein. In the canonical ubiquitination pathway, the C-terminal glycine of ubiquitin forms an isopeptide bond with the ε-amino group of a lysine residue—or, less commonly, with the N-terminal methionine—of the substrate protein [33]. It should be noted that in rare cases, ubiquitin can be attached to non-lysine residues of substrate proteins, such as serine, threonine, and cysteine (reviewed in [39]). During the sequential action of the enzymatic cascade, ubiquitin chains on the target protein can be elongated via formation of an isopeptide bond between the carboxyl group of the C-terminal glycine of one ubiquitin and the ε-amino group of a lysine residue on another ubiquitin. Ubiquitin contains seven lysine residues (Lys6, Lys11, Lys27, Lys29, Lys33, Lys48, and Lys63), which allow the formation of different ubiquitin linkage types, including seven homotypic and various heterotypic chains. Consequently, substrate proteins can be modified with diverse ubiquitin signals, such as monoubiquitination, multi-monoubiquitination, or polyubiquitin chains of varying lengths and linkage types (Figure 1).

Ubiquitinated molecules are subsequently recognized by other proteins containing ubiquitin-binding domains (ubiquitin receptors), including receptors associated with the proteasome or the autophagosome. The functional consequences of ubiquitination are determined by the type of ubiquitin modification. Polyubiquitin chains linked via Lys48 are most commonly associated with protein degradation by the proteasome. Lys11-linked polyubiquitin chains can also contribute to degradation, particularly in the context of cell cycle regulation [40]. Other types of ubiquitin chains perform functions unrelated to proteolysis. For example, Lys63-linked chains are involved in the regulation of signaling pathways (such as NF-κB), DNA repair, endocytosis, and intracellular transport [41]. Linear (Met1-linked) chains play an important role in cell death and immune signaling [42]. Monoubiquitination (including multi-monoubiquitination) regulate the cellular localization of proteins, their activity, or participation in protein–protein interactions [43].

Importantly, ubiquitination is a reversible modification. DUBs remove ubiquitin moieties from target proteins, altering their functional state or preventing their degradation. Within the proteasome, DUBs remove ubiquitin chains from substrates when they are committed to degradation [44].

Disruption of certain components of the UPS can contribute to the development of various diseases, including malignant neoplasms. Therefore, the identification of specific inhibitors of this system appears to be a promising avenue for therapeutic intervention. However, it is important to consider that the UPS is a key player in maintaining cellular homeostasis, and inhibiting its function can disrupt numerous vital processes. Existing UPS inhibitors include compounds that affect not only the proteolytic activity of the proteasome but also other components of the system [45]. The least specific inhibitors target the proteolytic activity of the proteasome and the E1 enzyme, exerting toxic effects that typically induce apoptosis proliferating cells are generally more sensitive to these agents. A more advanced strategy involves developing selective inhibitors of E3 ligases or DUBs, enabling targeted disruption of defined protein-substrate interactions or E3/DUB complexes [46].

While the functional activity of HMGB-family proteins and their transport within the cell are regulated by PTMs such as acetylation, phosphorylation, and changes in the redox status [10,47,48], their cellular abundance is also critically modulated by ubiquitination and subsequent degradation, primarily via the proteasome pathway, alongside other regulatory mechanisms such as autophagy and transcriptional control [49,50,51,52,53,54,55,56]. Thus, the identification of HMGB-specific E3 ligases or DUBs represents a promising direction for developing small molecules targeting a range of human pathologies, including cancer.

The UPS plays a crucial role in regulating processes associated with tumor initiation, metastasis, invasion, and factors influencing cancer prognosis. As noted above, proteins of the HMGB family are also involved in these processes. Numerous studies have demonstrated that their expression levels are substantially elevated in cancer cells compared to normal cells [57]. These finding support the potential of HMGB proteins as prognostic markers and therapeutic targets across a wide spectrum of malignancies, including gastrointestinal (esophageal, gastric) [58,59], breast and cervical [60,61], lung (non-small cell lung cancer and adenocarcinoma) [62,63], and urogenital (prostate, ovarian) cancers [64,65].

To search for HMGB-specific E3 ubiquitin ligases and DUBs, we used publicly available literature databases; search queries included the keywords listed on the title page of the article. The search was largely limited to articles published from 2010 onwards. E3 ligases and DUBs were included in Table 1 only if they had been demonstrated to directly ubiquitinate or deubiquitinate HMGB proteins. It should be noted that HMGB1 ubiquitination remains poorly characterized, while this PTM has been virtually unexplored for other members of the HMGB family. Consequently, most HMGB-specific E3 ubiquitin ligases and DUBs identified to date have been reported in association with HMGB1 (Table 1).

### 2.1. E3 Ubiquitin Ligases

E3 ligases, along with DUBs, are key components of the UPS. More than 600 E3 ubiquitin ligases have been identified in humans. These ligases constitute a diverse enzyme family that differs primarily in the mechanism by which ubiquitin is transferred from E2 conjugating enzymes to target proteins. The largest group of E3 ubiquitin ligases is the RING (really interesting new gene) finger (RNF) proteins. RNF ligases mediate the direct transfer of ubiquitin molecules from E2 enzyme to the protein substrate [81]. In contrast, ligases of Homologous to E6-AP C-Terminus (HECT) family operate via a two-step mechanism, forming a transient thioester bond between ubiquitin and a cysteine residue within the E3 ligase [82,83]. Ligases of a third family, the RING-between-RING (RBR), occupy an intermediate position and utilize both the RING- and HECT-type mechanisms of ubiquitin transfer [84,85].

#### 2.1.1. RNF Family

##### RNF125

Hu et al. have shown that RNF125 directly interacts with the HMGB1 protein in both mouse and human bronchial epithelial cells [53]. The DNA-binding Box B of HMGB1 plays a key role in this interaction, leading to the protein’s degradation via the UPS. In vitro experiments demonstrated that RNF125 ubiquitinates HMGB1 specifically at lysine residue 150 located within the Box B and targets it for proteasomal degradation. The RNF125/HMGB1 axis influences autophagy and oxidative stress regulation. Hypermethylation of the *Rnf125* gene promoter reduces RNF125 expression, resulting in decreased ubiquitination of HMGB1 and its stabilization within cells. Accumulation of HMGB1 in human bronchial epithelial cells enhances oxidative stress and autophagy [53].

##### RNF186

In non-alcoholic fatty liver disease, another E3 ligase, RNF186, regulates liver lipid phagocytosis, while HMGB1 inhibits liver lipogenesis and protects the liver from steatosis progression [86]. Du et al. have demonstrated that RNF186 binds cytoplasmic HMGB1 in liver cells [54]. Polyubiquitin chains are formed at Lys48 and Lys63 residues, leading to subsequent proteasomal degradation of HMGB1. Importantly, the release of HMGB1 protein from the nucleus to the cytoplasm induces lipophagy in liver cancer cells. Moreover, knockdown of RNF186 accelerates lipophagy by inhibiting the ubiquitination of cytoplasmic HMGB1 and stabilizing it. Thus, the authors point to the RNF186/HMGB1 axis as a promising therapeutic target for the prevention and treatment of non-alcoholic fatty liver disease in humans.

##### RNF219

The E3 ligase RNF219 also influences the HMGB1 translocation, although a direct interaction between the two proteins has yet been reported. Hwang et al. showed that in lipopolysaccharides (LPS)-treated cells, RNF219 knockdown results in enhanced extracellular release of HMGB1 [49]. The effect is associated with enhanced sirtuin 1 (SIRT1)-mediated acetylation of HMGB1 is directly depend on SIRT1 activity. During inflammatory response, RNF219 modulates the stability of SIRT1, thereby influencing HMGB1 acetylation and release. Collectively, these finding indicate that RNF219 indirectly regulates HMGB1 translocation through its control of SIRT1 [49].

#### 2.1.2. BRCA1

Breast cancer gene 1 (BRCA1), a RING domain-containing E3 ubiquitin ligase primarily known for its role in DNA damage repair, mediates the ubiquitination of HMGB3, directing it for proteasomal degradation [50]. Additionally, BRCA1 interacts with synaptotagmin 7 (SYT7), leading to reduced ubiquitination of HMGB3. Through its interaction with BRCA1, SYT7 modulates HMGB3 stability, thereby contributing to the progression of thyroid cancer [50].

#### 2.1.3. CHIP

Recent studies have shown that carboxyl terminus of Hsc70-interacting protein (CHIP), also known as STIP1 Homology And U-Box Containing Protein 1 (STUB1) interacts with HMGB1 [52,66]. The interaction between CHIP and HMGB1 promotes ubiquitination and subsequent proteasomal degradation of HMGB1. This process results in reduced aerobic glycolysis, proliferation, and invasion of endometriotic cells, thereby slowing the progression of endometriosis [52,66].

A similar mechanism was observed in spinal cord injury [66]. Specifically, CHIP was demonstrated to promote the ubiquitination and proteasomal degradation of HMGB1, thereby suppressing oxidative stress damage and apoptosis in nerve cells to mitigate spinal cord injury [66]. Thus, CHIP may be considered an inhibitory factor that is expressed at low levels in endometriosis and spinal cord injury and negatively correlated with HMGB1.

#### 2.1.4. SYVN1

Proteasomal degradation of HMGB1 is also promoted by the E3 ubiquitin ligase SYVN1, which exerts an anticancer effect in papillary thyroid cancer and esophageal squamous cell carcinoma [55,67]. SYVN1 was demonstrated to ubiquitinate HMGB1, targeting it for proteasomal degradation [55]. In esophageal squamous cell carcinoma, elevated HMGB1 expression was shown to be directly associated with the reduced activity of lysine-specific demethylase 4D (KDM4D) [67]. Downregulation of KDM4D promotes cancer cell proliferation and migration. Mechanistically, KDM4D enhances *SYVN1* gene transcription through demethylation of histone H3 at Lys9, thereby facilitating HMGB1 degradation. Collectively, these finding indicate that the KDM4D/SYVN1/HMGB1 axis regulates tumor cell growth, migration, and self-renewal through modulation of HMGB1 levels [67].

A similar mechanism was observed in diabetic retinopathy, both in vitro (high glucose model using human retinal microvascular endothelial cells) and in vivo (diabetes model in rats), Procyanidin, a polyphenolic compound known for its potential to alleviate diabetes-related complications, was shown to activate ATF1 (activating transcription factor 1). Mechanistically, ATF1 enhanced the transcription of SYVN1, promoting HMGB1 degradation through ubiquitination, and suppressed the HMGB1/TLR4 signaling pathway, leading to inhibition of pyroptosis and angiogenesis [68]. Thus, a decrease in HMGB1 levels alleviates inflammation in the retinal microvasculature, thereby contributing to the preservation of visual function.

#### 2.1.5. TRIM Family

Most proteins of the tripartite motif-containing (TRIM) family possess E3 ubiquitin ligase activity and are involved in protein quality control, innate immunity, apoptosis, autophagy, carcinogenesis, intracellular signaling, and development [87,88]. Mass spectrometry analysis identified TRIM28 as a potential E3 ligase for HMGB1 [51]. However, knockdown of TRIM28 in human hepatocellular carcinoma cells did not significantly affect HMGB1 protein levels. Instead, HMGB1 was found to directly interact with hematological and neurological expressed 1 (JPT1/HN1), a protein involved in cell cycle regulation and adhesion [89,90]. Notably, JPT1/HN1 is overexpressed was detected in several cancers, including thyroid [91], breast [92], prostate [89,93], and liver [51], indicating its role in tumorigenesis and progression. In prostate cancer, JPT1 promotes proteasomal degradation of the androgen receptor, thereby inhibiting androgen receptor signaling pathway [89,93]. In hepatocellular carcinoma cells, Wang et al. demonstrated that JPT1 knockdown accelerates HMGB1 degradation and enhances the anticancer efficacy of oxaliplatin [51]. Mechanistically, JPT1/HN1 stabilizes HMGB1 by preventing its ubiquitination and autophagolysosomal degradation of HMGB1 through direct interaction with TRIM28.

Recently, Zhou et al. demonstrated that another ligase of TRIM family, TRIM37, directly interacts with HMGB1 at peripheral nerve injury. TRIM37 promotes the ubiquitination and degradation of HMGB1, which leads to the inhibition of Schwann cell ferroptosis [69]. This study demonstrated the regulatory role of TRIM37 interaction with HMGB1, but the identification of the mechanisms of this interaction requires careful investigation.

#### 2.1.6. PRKN

Parkin (PRKN) is an E3 ubiquitin ligase primarily known for its role in mitophagy and Parkinson’s disease pathogenesis, has emerged as a critical regulator of HMGB proteins through non-proteolytic ubiquitination [70,71,94]. PRKN was demonstrated to directly ubiquitinate HMGB1 at Lys146 within the Box B via Lys48-linked chains [70]. This modification event does not target HMGB1 for proteasomal degradation. Instead, PRKN-mediated K146 ubiquitination enables the selective loading of HMGB1 into autophagy and mitochondria-derived large extracellular vesicles for intercellular communication.

Beyond its neurological role, PRKN emerges as a dual-mode tumor suppressor that both inhibits intrinsic tumor traits (metabolism and cell invasion altering mitochondrial dynamics) and reinvigorates CD8^+^ T-cell functions in the tumor microenvironment [71]. Notably, PRKN expression is epigenetically suppressed by promoter hypermethylation in various human cancers, including breast, prostate, pancreatic, and liver carcinomas, and this silencing correlates with reduced patient survival. Re-expression of PRKN, either via transient transfection or clinically approved DNA hypomethylating agents such as decitabine, stimulates a potent interferon (IFN) response in tumor cells [71].

Mechanistically, PRKN-induced release of HMGB1 activates IFN signaling in recipient cells, expanding CD8+ T-cell subsets with effector, self-renewal, and cytotoxic proper-ties [71]. This preferentially expanded CD8^+^ T cell subsets, thereby reprogramming an antitumor immune microenvironment PRKN also regulates HMGB1 release in the context of inflammatory pathologies. In pancreatic tumorigenesis models, loss of PINK1 or PRKN leads to accumulation of mitochondrial iron importers (SLC25A37 and SLC25A28), resulting in mitochondrial iron overload, inflammasome activation, and subsequent HMGB1 release [95].

Another link between PRKN and HMGB1 was revealed in a study investigating bone tissue repair following traumatic brain injury (TBI) combined with fractures. Increased expression of PRKN in bone tissue under such conditions promotes healing. Chen et al. showed that in a mouse TBI fracture model, the expression levels of several proteins—including PRKN, the serine/threonine kinase PINK1, and BECN1—were significantly increased [72]. The close association between PINK1 and PRKN drives structural remodeling of damaged or dysfunctional mitochondria, thereby inducing mitophagy and promoting the clearance of impaired organelles [96]. Upon mitochondrial injury, PINK1 accumulates on the outer mitochondrial membrane and becomes activated by phosphorylation, leading to the recruitment of PRKN to the outer mitochondrial membrane. Notably, suppression of PINK1 expression attenuates the pro-healing effect of HMGB1 on fracture repair. Chen et al. has further suggested that HMGB1 contributes to fracture healing by activating of the canonical PINK1/PRKN-mediated mitochondrial autophagy pathway [72]. Following TBI, elevated PINK1 and PRKN levels are accompanied by enhanced mitochondrial autophagy [97]. Thus, HMGB1 accelerates fracture healing after TBI through the activation of PINK1/PRKN-dependent mitochondrial autophagy.

The PRKN-mediated mechanism differs from other E3 ligases that regulate HMGB protein stability. Rather than promoting HMGB1 degradation, PRKN redirects this protein from pro-inflammatory cytosolic and/or nuclear localization to the extracellular space, where it can exert immunomodulatory functions. In cancer, PRKN-induced extracellular HMGB1 engages TLR4 and RAGE on immune cells, triggering immunostimulatory cytokine production and anti-tumor CD8^+^ T-cell activation. In contrast, in chronic inflammatory diseases such as diabetic retinopathy or pancreatic inflammation, sustained HMGB1 release (whether PRKN-dependent or independent) activates the same receptors but contributed to pathological outcomes, including chronic inflammation, pyroptosis, angiogenesis, and fibrosis. These contrasting outcomes appear to reflect differences in the duration, magnitude, and cellular source of HMGB1 release, as well as underlying immune status of the affected tissue, rather than differences in the signaling mechanism itself. Consequently, although therapeutic PRKN activation of the PRKN-HMGB1 axis represents a promising strategy for cancer immunotherapy, it could theoretically exacerbate chronic inflammatory response under certain conditions. However, PRKN deficiency has also been associates with uncontrolled HMGB1 release, suggesting that PRKN functions primarily as a regulator of HMGB1 homeostasis rather than an inherently beneficial or detrimental factor. Accordingly, PRKN activation is likely to be beneficial in cancer and may be neutral or protective in chronic inflammation settings, although direct validation in non-malignant models is still lacking.

#### 2.1.7. SMURF2

The E3 ubiquitin ligase SMAD ubiquitination regulatory factor 2 (SMURF2), a member of the HECT-type ligase family involved in TGF-β receptor signaling, plays an important role in modulating endothelial inflammation and atherosclerosis. SMURF2 is a key regulator of cellular processes associated with the UPS, particularly those related to signal transduction and cellular stress responses [98]. In human umbilical vein endothelial cells (HUVECs), SMURF2 has been shown to participate in oxidative stress-induced apoptosis and to reduce reactive oxygen species (ROS) production induced by hydrogen peroxide [99]. A recent study by Liang et al. demonstrated increased SMURF2 expression in endothelial cells within atherosclerotic lesions in mice and in unstable carotid plaques in humans [56]. This effect appears to be endothelial cell-specific and is associated with decreased plaque formation on blood vessel walls. Additionally, macrophage recruitment to plaque areas is increased, while the expression of cell adhesion molecules is decreased, resulting in reduced monocyte adhesion. The authors identified HMGB1 as a novel substrate of SMURF2 with the interaction being enhanced under inflammatory conditions. In addition to the HECT catalytic domain, SMURF2 contains a WW domain that mediates protein–protein interactions. The WW domain interacts with the Box A of HMGB1, promoting its Lys48-linked polyubiquitination and proteasomal degradation. Through facilitating HMGB1 degradation, SMURF2 also regulates TNF-α-induced monocyte adhesion and endothelial inflammation. Overall, these finding indicate that SMURF2 suppresses endothelial inflammatory responses, highlighting its protective role in vascular homeostasis and its therapeutic potential in limiting the progression of atherosclerosis.

### 2.2. DUBs

DUBs constitute a family of diverse enzymes that play key roles in numerous cellular processes. Their primary functions include removal of ubiquitin from protein substrates and the cleavage of ubiquitin chains. By releasing ubiquitin molecules, DUBs protect proteins from proteasome-dependent degradation [100] and regulate their activity, stability, and subcellular localization [74,101,102,103,104]. Approximately 100 DUBs have been identified in humans, where they modulate key signaling pathways such as NF-κB, PI3K/Akt/mTOR, and MAPK. Through these pathways, DUBs influence tumor development, neurodegenerative disorders, cardiovascular diseases, inflammation, and other pathological conditions [101]. Importantly, the effects of DUBs are multifaceted. For example, deubiquitination of protein with protective or anti-injury functions can inhibit cell death, whereas deubiquitination of pro-death mediators can accelerate cell death [105]. Based on sequence and structural characteristic, DUBs are classified into cysteine proteases, including members of the ubiquitin-specific protease (USP), ovarian tumor (OUT), ubiquitin C-terminal hydrolase (UCH), Machado–Joseph disease proteases (MJD) families and metalloproteinases, such as those of the JAMM family [106].

USPs constitute the largest family of DUBs which share structural similarities and interact with a variety of proteins. More than 50 USP family members have been identified in the human genome, which primarily recognize ubiquitin chains bonded through Lys48 and Lys63 [101,107]. Human USPs include a large catalytic core with six domains that containing amino acids required for catalysis—cysteine (domain 1), histidine (domain 5), and aspartate/asparagine residues (domain 6) [108]. Domains 3 and 4 contain a conserved Cys-X-X-Cys motif associated with the zinc finger domain ZnF. Additionally, USPs have other functional domains responsible for ubiquitin binding, such as ubiquitin associated (UBA) and ubiquitin-interacting motif (UIM), which facilitate interactions with ubiquitin and substrates [109,110]. The ZnF domain activates hydrolytic reactions and regulates enzyme activity, while the UBA and UIM domains are responsible for substrate binding [111,112].

USP7 (HAUPS)

USP7 (also known as HAUSP, herpes virus-associated protein) plays a key role in osteogenesis. On one hand, USP7 suppresses the Wnt/β-catenin signaling by stabilizing Axin, a key protein in this pathway, thereby inhibiting osteoblast differentiation under physiological conditions. On the other hand, inhibition of USP7 enhances Wnt/β-catenin signaling and promotes osteogenesis. Also, it is known that USP7 stabilizes lysine demethylase 6B (KDM6B) and Yes-associated protein 1 (YAP1), counteracting osteoporosis and promoting osteogenic differentiation via both the Wnt/β-catenin and Hippo pathways. Additionally, USP7 maintains the capacity of human bone marrow mesenchymal stem cell for differentiation into osteoblasts, adipocytes, and chondrocytes [113,114,115]. Beyond its role in bone biology, USP7 regulates the stability of the tumor suppressor protein p53 directly and via its negative regulator MDM2; both proteins, in turn, are associated with HMGB family members. In a study by Lin et al. [73], USP7 was shown to directly binds HMGB1, deubiquitinates it, and prevents its proteasomal degradation in osteoporosis. USP7-mediated deubiquitination of HMGB1 promotes osteoclast formation.

USP12

Studies have shown that multiple myeloma cells exhibit elevated levels of USP12, which deubiquitinates and stabilizes HMGB1. Knockdown of USP12 in these cells decreases HMGB1 expression and suppresses of HMGB1-mediated autophagy. Consequently, the USP12/HMGB1 axis has emerged as a potential diagnostic and therapeutic target for the treatment of human multiple myeloma [74].

USP13

Another member of the USP family, USP13, interacts with HMGB1, stabilizes it, and regulates its secretion through deubiquitination. USP13 also controls HMGB1 translocation from the nucleus to the cytoplasm: elevated USP13 levels enhance the interaction between HMGB1 and CRM1, which is required for HMGB1 cytoplasmic translocation [75].

USP15

Recently, USP15 was identified as another HMGB1-interacting partner that deubiquitinates and stabilizes HMGB1 in papillary thyroid cancer [76]. Although USP15 does not affect HMGB1 mRNA levels, *USP15* gene knockout markedly reduces HMGB1 protein abundance, indicating that USP15 regulates HMGB1 primarily through post-translational stabilization.

USP22

USP22 promotes various cellular processes, including cell proliferation and apoptosis. To elucidate the role of USP22 in podocyte injury associated with hypertensive nephropathy, Peng et al. screened for potential of USP22-interacting proteins and identified HMGB1, an inflammasome-associated factor, as a direct partner [77]. The interaction between USP22 and HMGB1 appears to play a crucial role in regulating inflammatory and fibrotic pathways in podocytes, with USP22 directly binding HMGB1 and stabilizes it via deubiquitination. This mechanism was confirmed in studies of angiotensin II (AngII)-induced nephropathy in both mouse and human podocytes. Increased USP22 expression was observed in kidney tissues from patients with hypertensive nephropathy, in AngII-treated mouse models, and in cultured podocytes. Both in vivo and in vitro, reduced USP22 expression leads to decreased HMGB1 levels and secretion, improved renal function, and attenuated pathological changes. Tall-like receptor (TLR4), which is regulated by the USP22-HMGB1 axis, may also contribute to podocyte injury and inflammatory responses.

### 2.3. Other Enzymes

In addition to E3 ligases and DUBs, other enzymes also participate in the regulation of HMGB protein stability.

#### 2.3.1. VCP

Another enzyme involved in the regulation of protein degradation is valosin-containing protein (VCP). Unlike E3 ligases or DUBs, VCP interacts with already ubiquitinated substrates in conjuncture with cofactors Ufd1-Npl4 or p47. This interaction triggers ATP hydrolysis and induces conformational changes in the substrate proteins. Pu et al. demonstrated that, following binding to VCP, ubiquitinated proteins are either recycled by DUBs or subjected to accelerated proteasomal degradation [78]. Heidelberger et al. further demonstrated that inhibition of VCP disrupts ubiquitination of numerous proteins at Lys6-linked ubiquitin chains [116].

In the same study, the authors reported that HMGB1 stability in hepatocellular carcinoma cells increases upon VCP overexpression [78]. Elevated VCP expression is associated with poorer patient survival, and during liver cancer progression, VCP acts as an oncogene that interacts with HMGB1 to activate the PI3K/AKT/mTOR signaling pathway [78].

#### 2.3.2. Abzymes

Abzymes are immunoglobulins that possess enzymatic activity. In addition to binding highly specific antigens, these antibodies can function as proteases and nucleases. Beyond to protease and nuclease activities, abzymes are also capable of catalyzing other reactions, including redox reactions and ester bond hydrolysis. Multiple studies have shown the presence of natural abzymes in the blood of patients with systemic lupus erythematosus that are capable of hydrolyzing DNA [79,117,118,119], RNA [120,121], microRNA [122], and certain proteins [79,123,124]. Melamud et al. demonstrated that IgG antibodies isolated from patients with systemic lupus erythematosus catalyze the hydrolysis of DNA and DNA-binding proteins, including histones and HMGB1 [79]. The authors propose that the HMGB1 is hydrolyzed within the Box A-domain. It is important to note that hydrolyzing antibodies may contribute to reduced immune responses; however, they can also target functionally critical molecules. For example, abzymes from patients with arrhythmogenic cardiomyopathy cleave N-cadherin and desmoglein 2, leading to impaired cardiomyocyte cohesion [125]. Notably, all reported catalytic activities of immunoglobulins have been observed in vitro, highlighting the need for further in vivo studies to determine their physiological relevance.

#### 2.3.3. Extracellular Proteases

As emphasized throughout this review, HMGB1 exhibit diverse functions that depend on its PTMs and cellular localization. For example, extracellular HMGB1 amplifies immune and proinflammatory responses [126,127,128,129]. Among the potential therapeutic strategies, proteolytic modulation of HMGB1 is being considered as an approach that may be particularly effective in modulating its proinflammatory activity [80,130].

Sowinska et al. demonstrated that proteases such as neutrophil elastase, cathepsin G, and matrix metalloproteinase 3 (MMP3), which accumulate in arthritic joints, can cleave HMGB1 in vitro [80]. Moreover, neutrophil elastase and MMP3 alter HMGB1 receptor-binding affinity by cleaving its acidic C-terminal region. In contrast, cathepsin G rapidly and completely degrades HMGB1, suggesting a potential mechanism for regulating extracellular HMGB1 function during inflammatory responses.

### 2.4. Context-Dependent HMGB Ubiquitination

A critical factor in targeting components of the UPS for HMGB regulation is the context-dependent nature of many E3 ligases and DUBs. The same enzyme can produce opposite functional outcomes depending on the tissue, disease state, and cellular microenvironment. For example, E3 ligase PRKN acts as a tumor suppressor in cancer by promoting the non-proteolytic, immunostimulatory release of HMGB1, thereby reinvigorating anti-tumor CD8^+^ T-cell response [71]. However, in chronic inflammatory conditions like diabetic retinopathy or pancreatic inflammation, sustained HMGB1 release (whether PRKN-dependent or independent) activates the same receptors (TLR4/RAGE) but leads to pathological outcomes, including chronic inflammation, pyroptosis, and fibrosis [72,131].

Similarly, SYVN1 and CHIP promote HMGB1 proteasomal degradation, which is beneficial in suppressing tumor growth or mitigating acute spinal cord injury [103,106,122,123], but their overactivation in other contexts could theoretically impair necessary HMGB1-mediated tissue repair or immune signaling.

This paradox arises because the functional outcome of HMGB ubiquitination is determined not only by the enzyme itself, but by the duration and magnitude of HMGB1 release, the baseline immune status of the tissue, and the specific PTMs (e.g., acetylation, oxidation, and phosphorylation) which dictates the affinity of HMGB proteins for these enzymes. Therefore, therapeutic strategies targeting HMGB-regulating E3 ligases or DUBs cannot be considered as universally beneficial or harmful. Future drug development should utilize a context-dependency approach, prioritizing tissue-specific delivery systems or conditional modulators to avoid exacerbating comorbidities while simultaneously addressing the primary pathology.

## 3. Conclusions

For years, it has been assumed that members of the HMGB-family perform largely similar functions due to their highly conserved amino acid sequences. However, recent evidence indicates that their expression patterns, interacting partners, and regulatory mechanisms differ substantially. Alterations in HMGB protein expression and localization are early diagnostic markers and predictors of disease severity across a wide spectrum of pathologies, from cancer to neurodegenerative and autoimmune disorders.

This review has summarized current knowledge regarding the E3 ubiquitin ligases and DUBs that regulate HMGB proteins’ stability. While the UPS is a critical post-translational regulator of HMGB abundance, it is important to emphasize that it is not the only control mechanism. HMGB levels and activity are regulated by a multilayered regulatory network that includes transcriptional control, various PTMs (such as acetylation, methylation, phosphorylation, and redox modifications), non-classical secretory pathways (e.g., extracellular vesicle release), and autophagy–lysosomal degradation. Therefore, ubiquitination should be considered as an important, context-dependent regulatory mechanism that fine-tunes HMGB protein function.

Furthermore, as highlighted in this review, enzymes such as PRKN, SYVN1, and CHIP exhibit profound context-dependency, acting as tumor suppressors in malignancies while potentially exacerbating chronic inflammation or fibrotic diseases in other tissues. This “two-pronged” nature means that systemic inhibition or activation of these targets carries significant risks. Future therapeutic strategies must move beyond broad UPS inhibition and focus on developing highly specific, tissue-targeted modulators or PROTACs that can selectively correct HMGB activity in a disease-specific manner. Exploring these nuanced regulatory networks offers promising, albeit complex, therapeutic opportunities for a wide range of human pathologies.

## Figures and Tables

**Table 1:** Enzymes involved in the degradation of HMGB proteins.

Ferment	Protein	Description	References
E3 UBIQUITIN LIGASES			
RNF125	HMGB1	RNF125 directly interacts with the HMGB1-protein in both mouse and human bronchial epithelial cells The RNF125/HMGB1 axis influences autophagy and oxidative stress regulation.	[53]
RNF186	HMGB1	RNF186 binds cytoplasmic HMGB1 in liver cells, leading to subsequent proteasomal degradation of HMGB1.	[54]
RNF219	HMGB1	RNF219 indirectly regulates HMGB1 translocation through its control of SIRT1.	[49]
BRCA1	HMGB3	BRCA1 interacts with synaptotagmin 7 (SYT7), which reduces HMGB3 ubiquitination. Through the BRCA1-SYT7 interaction, SYT7 stabilizes HMGB3, thereby promoting thyroid cancer progression.	[50]
CHIP	HMGB1	CHIP mediates HMGB1 ubiquitination and promotes its proteasomal degradation, thereby reducing endometriosis-associated glycolysis, proliferation, and invasion. In spinal cord injury, HMGB1 regulation is linked to inflammatory signaling and tissue damage.	[52,66]
SYVN1	HMGB1	SYVN1 ubiquitinates HMGB1, targeting it for proteasomal degradation, thus regulating tumor cell growth, migration, and self-renewal in papillary thyroid cancer and esophageal squamous cell carcinoma. SYVN1-mediated ubiquitination and proteasomal degradation of HMGB1 suppress HMGB1/TLR4 signaling, thereby inhibiting pyroptosis and angiogenesis in diabetic retinopathy.	[67,68]
TRIM28	HMGB1	HMGB1 stabilization is mediated by interaction with TRIM28, facilitated by hematological and neurological expressed 1 (JPT1/HN1), thereby preventing its ubiquitination and autophago-lysosomal degradation.	[51]
TRIM37	HMGB1	TRIM37 directly interacts with HMGB1, promoting its degradation, leading to the inhibition of Schwann cell ferroptosis.	[69]
PRKN	HMGB1	Ubiquitination occurs at Lys146 of HMGB1 within the Box B via Lys48-linked chains. This modification results in the release of HMGB1 promotes into extracellular space, which ultimately leads to reprogramming antitumor immunity via stimulation of IFN signaling and expansion of specialized CD8^+^ T cell subsets. HMGB1 contributes to fracture healing by activating the canonical PINK1/PRKN-mediated mitochondrial autophagy pathway.	[70,71,72]
SMURF2	HMGB1	SMURF2 interacts with the Box A, promoting its Lys48-linked polyubiquitination and proteasomal degradation of HMGB1.	[56]
DEUBIQUITINASES (DUBs)			
USP7	HMGB1	USP7 directly binds HMGB1, deubiquitinates it, and prevents its proteasomal degradation in osteoporosis. USP7-mediated deubiquitination of HMGB1 promotes osteoclast formation.	[73]
USP12	HMGB1	USP12 deubiquitinates and stabilizes HMGB1, leading to autophagy, improving survival in multiple myeloma.	[74]
USP13	HMGB1	USP13 interacts with HMGB1, stabilizes it, and controls HMGB1 translocation from the nucleus to the cytoplasm.	[75]
USP15	HMGB1	USP15 deubiquitinates and stabilizes HMGB1 in papillary thyroid cancer.	[76]
USP22	HMGB1	USP22 directly binds HMGB1 and stabilizes it via deubiquitination. The interaction may play a crucial role in regulating inflammatory and fibrotic pathways in podocytes.	[77]
OTHER ENZYMES			
VCP	HMGB1	VCP directly interacts with HMGB1, decreasing its degradation via proteasome and acts as an oncogene by activating the PI3K/AKT/mTOR signaling pathway during liver cancer progression.	[78]
Abzymes	HMGB1	IgG antibodies isolated from patients with systemic lupus erythematosus catalyze the hydrolysis of DNA and DNA-binding proteins, including histones and HMGB1.	[79]
Extracellular proteases	HMGB1	Extracellular proteases such as neutrophil elastase, cathepsin G, and metalloproteinase 3 (MMP3), which accumulate in arthritic joints, can cleave HMGB1 in vitro.	[80]

## Data Availability

No new data were created or analyzed in this study. Data sharing is not applicable to this article.
